# Impact of smoking cannabidiol (CBD)-rich marijuana on driving ability

**DOI:** 10.1080/20961790.2021.1946924

**Published:** 2021-09-28

**Authors:** Tim J. Gelmi, Wolfgang Weinmann, Matthias Pfäffli

**Affiliations:** a Department of Forensic Toxicology and Chemistry, Institute of Forensic Medicine, University of Bern, Bern, Switzerland; b Graduate School for Cellular and Biomedical Sciences (GCB), University of Bern, Bern, Switzerland; c Department of Traffic Sciences, Institute of Forensic Medicine, University of Bern, Bern, Switzerland

**Keywords:** Forensic sciences, forensic toxicology, cannabidiol, driving while impaired, road safety, driving assessment

## Abstract

To investigate effects of smoking cannabidiol (CBD)-rich marijuana on driving ability and determine free CBD and Δ^9^-tetrahydrocannabinol (THC) concentrations in capillary blood samples, a randomised, double-blind, placebo-controlled, two-way crossover pilot study was conducted with 33 participants. Participants smoked a joint containing 500 mg of tobacco and either 500 mg of CBD-rich marijuana (16.6% total CBD; 0.9% total THC) or 500 mg of a placebo substance, then performed three different dimensions of the Vienna Test System TRAFFIC examining reaction time, behaviour under stress, and concentration performance. For further assessment of participants’ fitness to drive, three tests of balance and coordination were evaluated and vital signs (blood pressure and pulse) were measured. Dried blood spot samples of capillary blood were taken after smoking and after completion of the tests to determine the cannabinoid concentrations (CBD, THC and THC-metabolites). The results revealed no significant differences between the effects of smoking CBD-rich marijuana and placebo on reaction time, motor time, behaviour under stress, or concentration performance. Maximum free CBD and THC concentrations in capillary blood were detected shortly after smoking, ranging between 2.6–440.0 ng/mL and 6.7–102.0 ng/mL, respectively. After 45 min, capillary blood concentrations had already declined and were in the range of 1.9–135.0 ng/mL (free CBD) and 0.9–38.0 ng/mL (free THC). Although the observed levels of free THC concentrations have been reported to cause symptoms of impairment in previous studies in which THC-rich marijuana was smoked, no signs of impairment were found in the current study. This finding suggests that higher CBD concentrations cause a negative allosteric effect in the endocannabinoid system, preventing the formation of such symptoms. Nevertheless, it is recommended that consumers refrain from driving for several hours after smoking CBD-rich marijuana, as legal THC concentration limits may be exceeded.

Supplemental data for this article is available online at https://doi.org/10.1080/20961790.2021.1946924 .

## Key points

- No significant impact on driving ability was found after smoking CBD-rich marijuana.
- No effects on vital signs were observed after smoking CBD-rich marijuana.
- All participants exceeded the Driving Under the Influence of Drugs (DUID) legal limit for THC in blood after smoking CBD-rich marijuana.

## Introduction

Cannabis containing a Δ^9^-tetrahydrocannabinol (THC) level exceeding 1% is listed as a prohibited narcotic in Switzerland, and, besides alcohol, is the most frequently abused drug in Switzerland and worldwide [1]. The results of an addiction monitoring survey [2] in 2016 revealed that one-third of the Swiss population aged 15 years and over had experience using cannabis as a drug. On the basis of this finding, it can be estimated that approximately 220 000 people consume cannabis in Switzerland.

THC is well known for its psychotropic properties as a drug of abuse, but also for its medical applications. Cannabidiol (CBD) is the second-most abundant and therapeutically most relevant medi­cinal component of cannabis [3, 4]. CBD has received significant interest over the last several years because of its reported pharmacological effects in a range of conditions, from inflammatory and neurodegenerative diseases [5], to epilepsy [6–14], autoimmune disorders like multiple sclerosis [15–18], schizophrenia [19–22] and cancer [23, 24]. However, there is currently insufficient toxicological and clinical data regarding these therapeutic effects [25, 26]. Moreover, CBD is considered to regulate and alleviate THC-related adverse effects, including tachycardia, anxiety, sedation and hunger [27, 28]. CBD is not associated with psychoactivity and does not affect motor function, memory, heart rate, blood pressure or body temperature alone [29]. However, Zuardi et al. [30] reported that, in mixed use of CBD and THC, the time between uptake of CBD and THC and the CBD/THC ratio appear to play important roles in the interaction between the two cannabinoids. Thus, CBD may increase the effects of THC through pharmacokinetic interaction if it is consumed before THC. However, a reverse interaction may occur when both cannabinoids are taken together, particularly at a high dose ratio of CBD/THC [30].

Since 2017, products containing CBD have been increasingly available on the Swiss market. Whereas only five companies were registered with the Federal Customs Administration at the beginning of 2017, one and a half years later (July 2018) there were 630 registered companies [31]. Tobacco substitute products with low THC (<1% total THC) and high CBD levels have received particular interest. This type of cannabis does not fall under the Federal Act on Narcotics and Psychotropic Substances (NarcA, 812.121) and the Ordinance of the Federal Department of Home Affairs on the lists of narco­tics, psychotropic substances, precursors and auxiliary chemicals (NarcO-FDHA, 812.121.11). Although there are currently no statistics on the consumption behaviour of adults, a recent study by Delgrande Jordan et al. [32] reported that almost 10% of boys and 5% of girls aged 15 years had consumed CBD. Products containing CBD-rich marijuana can be legally acquired not only in headshops, but also in supermarkets. However, due to the traces of THC, the consumption of CBD-rich marijuana can lead to detectable THC blood concentrations that violate the Federal Act on Road Traffic (RTA, 741.01) and the Ordinance on Traffic Regulations (TRO, 741.11). In addition to other jurisdictions in Europe and worldwide, Switzerland has adopted a zero tolerance policy for Driving Under the Influence of Drugs (DUID). This implies that, if the total THC concentration is above a decision limit (defined as 1.5 ng/mL in whole blood for analytical reasons) the driver has committed an offence, whether or not they are actually impaired. In forensic practice, a confidence interval of ±30% is taken into account due to measurement uncertainty. This results in an analytically determined THC concentration limit of 2.2 ng/mL (2.2 ng/mL minus 30% is 1.54 ng/mL, which is just above the legal decision limit of 1.5 ng/mL for zero tolerance).

The first pilot study on the pharmacokinetics of CBD was conducted in Switzerland in 2017, in which a voluntary subject smoked one, two, or up to four CBD joints within a short time period. The results showed that THC concentrations in blood of up to 6.8 ng/mL occurred after smoking CBD-rich marijuana, but, after less than 1 h, these concentrations had already fallen below the cut-off value of 2.2 ng/mL for determining incapacity to drive [33]. Another published pilot study by Meier et al. [34] also confirmed that after smoking CBD-rich marijuana, THC concentrations in the blood exceeded the Swiss legal limit, approaching 5 ng/mL in some cases. No accumulation was observed when consuming two joints per day on several consecutive days.

In a recent study by Spindle et al. [35], participants performed computerised cognitive and psychomotor tasks that can be generalised to operating a motor vehicle, after consuming oral and vaporised CBD-rich marijuana. Furthermore, several studies have examined driving ability after consumption of THC-rich marijuana. These previous studies can be divided into three types: those evaluating the effects at the psychomotor and/or neurocognitive levels under laboratory conditions [36–38], those examining simulated driving performance [39] and those investigating the direct effects on driving ability in highway tests [40–42].

Because of the legal availability of CBD-rich mari­juana and consumer behaviour in Switzerland, we sought to conduct a pilot study to investigate the impact of smoking CBD-rich marijuana on a range of psychomotor and neurocognitive skills associated with driving ability. The purpose of the current study was to inform recommendations for warnings on tobacco substitute products containing CBD-rich marijuana and to provide information for drivers regarding the possible risks of consuming CBD-rich marijuana. To the best of our knowledge, the current study is one of the first to investigate the potential impact of smoking CBD-rich marijuana for road safety.

## Experimental procedures

### Participants

The study was approved by the Ethics Committee of the Canton of Bern, Switzerland (BASEC-ID 2018-02301). Thirty-three volunteers (age range: 19–31 years, male: 19; female: 14) participated in this study. Participants were recruited through public advertisement and selected on the basis of their smoking behaviour and their availability. Only smo­kers or persons with smoking experience were included in the study. Experience with smoking CBD- or THC-rich marijuana was not inclusion criterion. All participants declared that they did not use other drugs of abuse (not including abstinence from alcohol or tobacco) and that they had abstained from cannabis use for at least 3 weeks before the examination. See Supplementary Table S1 for further details on the demographics of the participants.

**Table 1.** Statistics results of testing for significant differences in the traffic psychological assessment.

Groups	*P*-value			
	Reaction time	Motor time	Determination	Cognitrone
CBD *vs.* placebo	0.838	0.726	0.900	0.627
Women *vs.* men				
Placebo	0.453	0.004*	0.869	0.250
CBD	0.044*	0.004*	0.779	0.128
1st trial *vs.* 2nd trial	0.928	0.318	0.320	0.112

* Values marked with an asterisk are considered significant; CBD: cannabidiol.

Participants were excluded if they had a history of drug abuse (according to self-report data), known hypersensitivity to cannabinoids, were pregnant, suffered from infectious, metabolic, ear/nose/throat, autoimmune, respiratory, cardiovascular, psychiatric or neurological diseases, cancers, liver or kidney dysfunction or cognitive impairment, appeared to be inebriated on test days or were considered pain patients.

Each volunteer was informed about the possible risks and signed an informed consent form confirming that, to the best of their knowledge, none of the above-mentioned exclusion criteria applied to them.

### Design and procedures

#### Design

This single-centre pilot study, set up as a randomised, double-blind, placebo-controlled, two-way crossover study, was an initial investigation of the influence of smoking CBD-rich marijuana on driving ability. After smoking a joint containing either CBD-rich marijuana (test group) or a placebo (control group), psychomotor and neurocognitive tests were performed to determine the influence on driving ability.

#### Cannabis and placebo

Participants smoked either a CBD joint containing marijuana with a total CBD concentration of 16.6% (w/w) and a total THC concentration of 0.9% (w/w), or a placebo joint, containing a product called Knaster Hemp, a nicotine- and cannabinoid-free herbal mixture with a hemp aroma. Cangenus AG (Bachenbülach, Switzerland) provided the CBD-rich marijuana and Knaster Hemp was ordered online *via* Chillhouse GmbH (Röhrsdorf, Germany). The cannabinoid profiles of the CBD-rich marijuana and the placebo were determined using an updated high-performance liquid chromatography combined with UV detection (HPLC-UV) method [43]. The joints consisted of conically pre-rolled shells made of ultra-thin rolling paper with an integrated cardboard filter (Vandenberg Special Products B.V., Rotterdam, the Netherlands). Each shell was filled with 500 mg of additive free tobacco (Fred & Fly, Lausanne, Switzerland) and 500 mg of either CBD-rich marijuana or Knaster Hemp (mean weight ± SD per joint: (999 ± 22) mg). This resulted in doses of 83 mg of CBD and 4.5 mg of THC per CBD joint. Participants were not required to follow any standardised smoking procedure, but were instructed to smoke the joint *ad libitum* within 10 min.

#### Tasks

##### Traffic-related psychological assessment

To assess the participant’s fitness to drive, three different dimensions of the Vienna Test System TRAFFIC (Schuhfried GmbH, Moedling, Austria) were evaluated. This system allows measurement and examination of attitudes relevant to road safety with validated and standardised tests, and is designed to aid reliable decision-making regarding an individual’s fitness to drive. This system is currently used in 26 countries, and its standardised and objective evaluation method ensures that the same conditions apply to all participants, regardless of their cultural background or level of education (Schuhfried-GmbH, available from: www.schuhfried.com).

First, the reaction test (RT) of the Vienna Test System was performed. Participants were instructed to react as quickly as possible to visual and acoustic signals. This involved pressing or releasing a button as quickly as possible when two stimuli were presented simultaneously (yellow light and tone). The use of a rest button and a reaction button allowed the data to be divided into reaction time and motor time.

Second, participants’ behaviour under stress was tested. The determination test (DT) was used to measure the reactive resilience, or the ability to react under complex stimulus conditions. In the DT para­digm, buttons and foot pedals can be used to react to both coloured stimuli and acoustic signals. The stress element in the DT emerges in responding as quickly and accurately as possible to rapidly changing stimuli. The adaptive test specification allows each participant to be put in an overstrained situation with an adequately high stimulus frequency, so that they can no longer perform the required reactions. This makes it possible to study behaviour under varying degrees of psychophysical stress (Schuhfried-GmbH, available from: www.schuhfried.com).

Third, participants’ concentration performance was tested. In the Cognitrone test, the participant compares one geometric figure with four other geometric figures. The participant then indicates whether the comparison figure corresponds to one of the other four geometric figures. In the current study, a test format with free processing time was used, in which participants were instructed to press different keys to indicate whether the figure was identical to another figure or not (Schuhfried-GmbH, available from: www.schuhfried.com).

##### Tests of balance and coordination

For further assessment of participant’s fitness to drive, we used three tests for balance and coordination based on the Drug Recognition Experts (DRE) programme [44] and Hauri-Bionda et al. [45]. These tests are regularly used in Switzerland by trained medical personnel on behalf of the police to determine neurological deficits after substance misuse and/or possible intoxication of persons suspected of impaired driving.

First, the participant’s balance was tested using the Romberg test, combined with his/her internal clock. The participant was asked to stand upright with his/her feet together. The participant stretched his/her arms out horizontally with the palms of the hands facing upwards, the fingers spread and the head slightly reclined. As soon as the participant felt ready, he/she closed his/her eyes and estimated a time span of 30 s. The participant opened his/her eyes again to signal that he/she felt the time span was over. Meanwhile, the examiner measured the real time span, from the moment the participant closed his/her eyes to the moment he/she opened his/her eyes.

Second, the participant’s coordination was tested using the Finger-to-Nose test. The participant was asked to stand upright with his/her feet together. The participant was instructed to stretch his/her arms out to the side of the body with extended index fingers, with the head slightly reclined and the eyes closed. The main task was to use alternate arms to touch the tip of the nose with the extended index finger, then bring the arm back into the starting position. The sequence performed by each participant was left-right-left-right-right-left.

**Figure 1.**
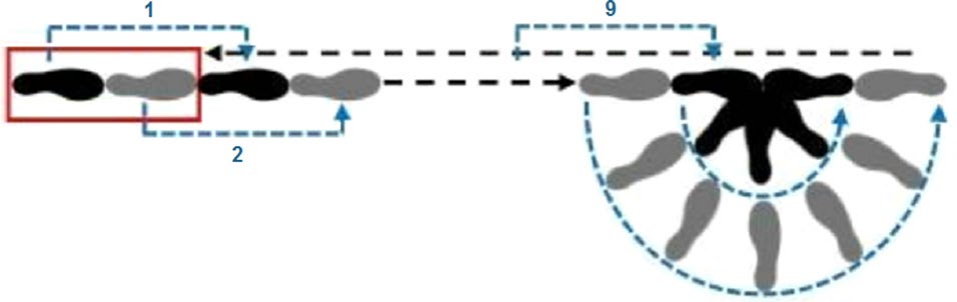
Execution of the Walk-and-Turn test (red rectangle: starting position; black: left foot; grey: right foot). 1: the first step; 2: the second step; 9: the ninth step.

Third, the participant’s balance and coordination were tested using the Walk-and-Turn test. The participant was asked to place his/her left foot first, then to place his/her right foot on the line, so that the heel of the right foot touched the tip of the left foot. The participant was instructed to take nine steps on the line by placing one foot in front of the other so that the heel of the front foot touched the tip of the back foot. The participant looked at his/her feet and counted each of the steps aloud. A 180° turn of the body was then performed, with the front foot always remaining on the line, while the other foot made small steps to turn. After the rotation, the front foot was placed in front again and the rear foot touched the heel of the front foot with its tip. In the same way as before, the participant then took nine steps forward (Figure 1).

##### Vital signs and observable changes in behaviour, orientation, mood, language and psychomotor skills

The vital signs (blood pressure and pulse) were measured with an automatic blood pressure device (BM 28; Beurer GmbH, Ulm, Germany) before the tests of balance and coordination. In addition, the psychologist paid attention to any changes in behaviour, orientation, mood, language or psychomotor skills during the tests.

#### Procedure

On the first test day, a coin was tossed to randomise participants into two groups. Depending on which side the coin landed, either a joint with CBD-rich marijuana or placebo was handed to participants. The participant then smoked the joint within 10 min and was taken to the first examination room. An initial blood sample was taken before testing, followed by the traffic psychological assessment, recording of vital signs and tests of balance and coordination. After the last test, another blood sample was taken. The procedure on the second test day (7 to 14 days later, with an average of 9 days) was identical to the procedure on the first test day, but without the coin toss. Participants who had smoked a CBD joint on the first day of testing received a placebo joint and *vice versa*. The average total test time between the two blood samples on the first test day was slightly longer ((48 ± 5) min) than that on the second test day ((42 ± 6) min) because the tests needed to be explained on the first day, whereas participants knew what to expect on the second day of testing.

### Evaluation and data analysis

#### Traffic psychological assessment

The Vienna Test System TRAFFIC provides validated tests in a traffic psychology setting that are relevant to road safety. Furthermore, this test provides easily understandable results and fully automated evaluations, individual reports, detailed profiles and comprehensive overall assessments. The measured test variables are first output as raw values. However, the raw values regarding a participant’s competence are meaningless without knowing how other partici­pants usually perform in the same test [46]. Thus, these raw values are compared to an extensive representative norm sample compiled by Schuhfried GmbH from data collected in German-speaking countries. This process results in a percentage rank (PR) value, which indicates the percentage of the reference population that performed at least as well as the tested participant. In the evaluation of PR values and the presentation of a participant’s profile, a distinction is made between below average (PR < 16), average (16 ≤ PR ≤ 84) and above average (PR > 84). In principle, a PR of 16 should be achieved or exceeded in all performance tests concerning driving ability, whereby isolated results below this criterion can be considered compensable (e.g. through driving experience, insight into existing deficits, prudent behaviour) [47, 48].

In the reaction test, the following variables were evaluated:

- Average reaction time: measurement of the time between the beginning of the display of the required stimuli and the release of the rest button. A high PR value indicates that, compared with the reference population, the participant has an above-average ability to respond quickly and appropriately to relevant stimulus constellations (Schuhfried-GmbH, available from: www.schuhfried.com).
- Average motor time: measurement of the time between the release of the rest button and the contact with the reaction button when the relevant stimuli are presented. This variable provides information about the speed of movement of the participant. A high PR value indicates that, compared with the reference population, the participant has an above-average ability to quickly implement appropriately planned action sequences in reaction situations (Schuhfried-GmbH, available from: www.schuhfried.com).

The main variable assessed in the DT was as follows:

- Correct: indicates the number of all correct reactions performed by the start of the next but one stimulus at the latest. This evaluation measured the ability of the participant to continue to react quickly and adequately in reaction chains, even in the range of the participant’s individual stress limit (Schuhfried-GmbH, available from: www.schuhfried.com).

The main variable assessed in the Cognitrone test was as follows:

- Average time of “correct rejection”: measurement of selective attention in terms of the energy required to maintain a certain level of accuracy. Participants performing well on this variable are characterised by a high level of concentration. This indicates that the participant’s ability to focus his/her attention on relevant information is highly developed, exhibiting a quick working style when performing in a concentrated manner (Schuhfried-GmbH, available from: www.schuhfried.com).

**Figure 2.**
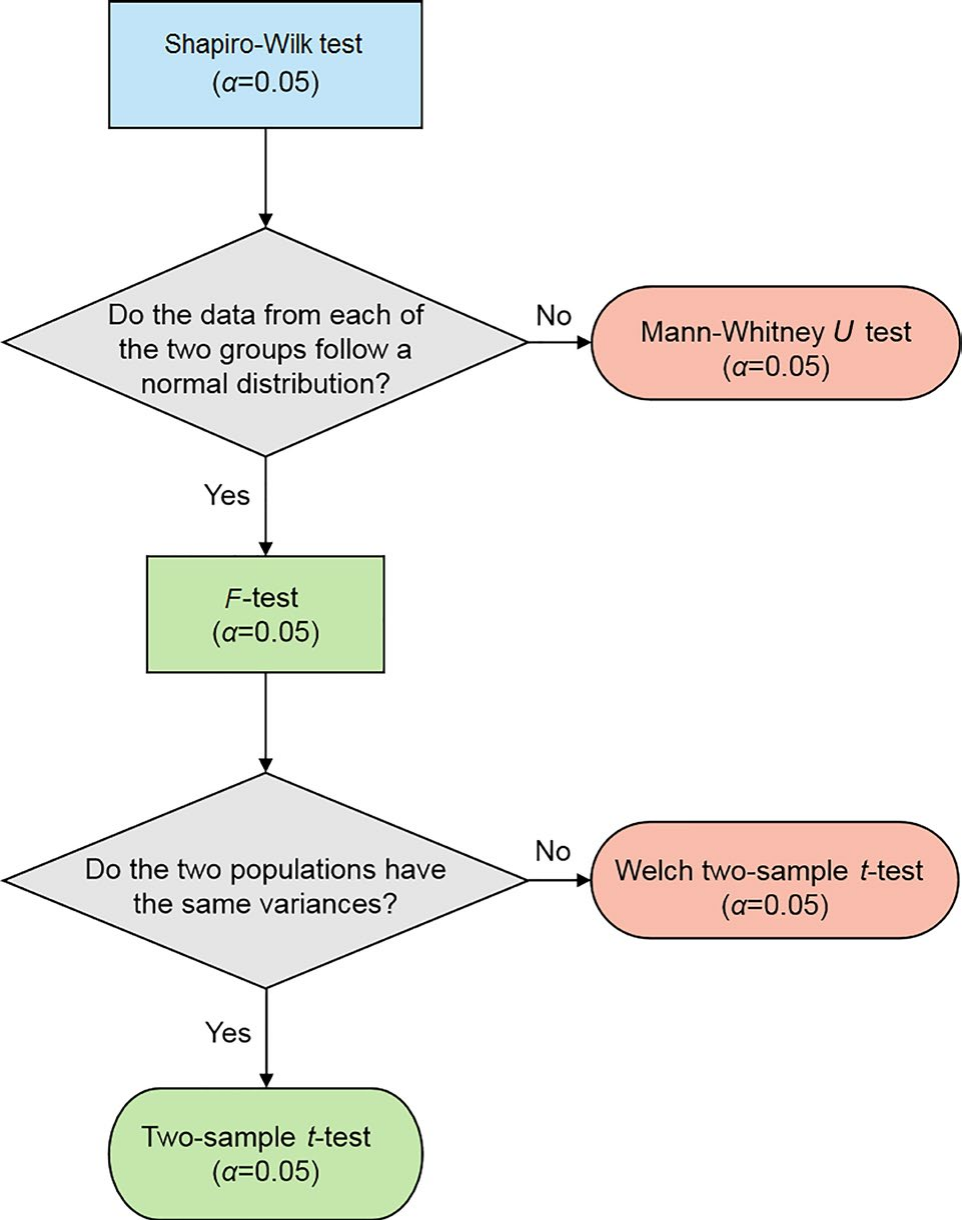
Statistical evaluation plan to determine significant differences in mean values.

A problem with PR values is that differences (contrary to intuitive presumptions) cannot be interpreted reasonably because they are rank- but not interval-scaled. For example, it is not possible to say that an improvement from a PR 2 to a PR 12 (10 PR difference) is worth as much as an improvement from PR 50 to PR 60 (although both involve a 10 PR difference) [46]. Thus, to statistically eva­luate performance differences in traffic psychological assessments, the percentage ranks were converted into standard *t*-values using the table reported by Lauth et al. [49]. The statistical evaluations and visualisations were carried out using Excel (Microsoft, Redmond, WA, USA) and R version 3.6.2 (The R Foundation). To determine whether significant differences in mean values existed, different tests were performed, selected according to the scheme indicted in Figure 2. The following groups were distinguished: CBD *vs.* placebo consumption; women *vs.* men and 1st trial *vs.* 2nd trial. A significance level of *α*=0.05 was set for all tests, and the null hypo­thesis (*H*0) was defined in such a way that the mean values of both groups were equal (*H*0: mA=mB).

#### Tests of balance and coordination

During the Romberg test, we recorded whether the participant had stable balance, whether he/she swayed strongly, or whether he/she was unable to perform the test. Furthermore, we noted whether the participant opened his/her eyes before the end of the test and how many seconds he/she estimated as 30 s. These time spans were statistically evaluated and visualised using Excel and R version 3.6.2 (Figure 2).

During the Finger-to-Nose test, we recorded whether the participant touched the tip of his/her nose or not. Furthermore, we noted whether the movements were undisturbed or whether zigzag movements or intention tremor were observed. In addition, we assessed whether the motion sequence (left-right-left-right-right-left) was correct or incorrect.

During the Walk-and-Turn test, we recorded whether the participant exhibited interrupted walking, whether the heel-to-toe was missed, whether the participant was hopping around and whether the rotation was performed correctly. The number of steps taken was also noted.

#### Vital signs and observed changes in behaviour, orientation, mood, language and psychomotor skills

Vital signs were visualised using Excel and R version 3.6.2. Observations of changes in behaviour, orientation, mood, language and psychomotor skill were registered and evaluated by a psychologist.

### Blood sampling and sample analysis

After thoroughly washing the hands and disinfecting the fingertips, the first capillary blood sample was taken approximately 5 min after finishing smoking and a second sample was taken after completing all tests (approximately 45 min later). For sampling, the side of the fingertip was pricked with a sterile, single-use lancing device (ACCU-CHECK® Safe-T-Pro Plus; Roche, Mannheim, Germany) and 20 μL of blood was taken in quadruplets with disposable micropipettes end to end 20 μL (Minicaps®; Hirschmann® Laborgeräte GmbH & Co. KG, Eberstadt, Germany). The samples were transferred directly to filter paper (grade 903, CF12; Whatman^®^, Uppsala, Sweden) and left to dry for at least 2 h at ambient temperature. Afterwards, the samples were stored in the freezer at −25 °C in Minigrip^®^ bags containing a silica gel desiccant bag (PROPASIL^®^; Propagroup S.p.A., Rivoli, Italy) and analysed within 1 week.

Sample preparation was based on an internally validated extraction method and was adapted for analysis of cannabinoids. Whole dried blood spots were punched out (10 mm i.d.) and transferred to an Eppendorf tube. For each sample, 1 mL of acetonitrile (HPLC gradient grade, 99.9%; Acros Organics, Geel, Belgium) and 10 μL of internal standard (THC-*d3*, CBD-*d3* and 11-OH-THC-*d3* at 0.1 μg/mL and THC-COOH-*d3* at 0.5 μg/mL; all from Cerilliant, Round Rock, TX, USA) were added. After shaking for 10 min and centrifugation for 10 min with 13 000 rpm at 8 °C, the solvent was transferred into a micro-vial. The solvent was then evaporated to dryness under a gentle stream of nitrogen and reconstituted in 100 μL of acetonitrile/water/formic acid (60/40/0.1; *v/v/v)*. Aliquots of 25 μL were then injected into the liquid chromatography-tandem mass spectrometry (LC-MS/MS) system.

Cannabinoid concentrations were determined using our previously published column-switching on-line SPE LC-MS/MS method [33, 50], modified using a newer, more sensitive mass spectrometer. The LC-MS/MS consisted of an UltiMate 3000 HPLC system (Dionex, Olten, Switzerland), coupled to a 5500 QTRAP hybrid triple quadrupole/linear ion trap mass spectrometer with a Turbo V ion source (SCIEX, Brugg, Switzerland). Analyst software version 1.6.2 (SCIEX) was used for data acquisition and analysis.

## Results

### Traffic psychological assessment

#### Reaction test

The *t*-values for average reaction time ranged from 46.5–73.0 (mean: 60.6) after consumption of the CBD joints and from 46.5–77.5 (mean: 61.0) after consumption of the placebo joints. The average *t*-values for motor time ranged from 38.0–77.5 (mean: 55.0) and from 38.0–69.0 (mean: 55.7) after consumption of the CBD joints and placebo joints, respectively. There were no significant differences in reaction time or motor time between smoking CBD-rich marijuana and placebo. Whereas there was no significant gender difference in reaction time after smoking the placebo, there was a significant gender difference after smoking CBD (*P* = 0.004). Furthermore, there were significant gender differences in motor time after smoking, in both the placebo (*P* = 0.004) and CBD-rich (*P* = 0.004) marijuana conditions. The analysis revealed no significant differences between the first and second day of testing. Learning effects can therefore be excluded (Table 1).

#### Determination test

The *t*-values for the total number of correct reactions ranged from 37.5–77.5 (mean: 60.9) after smoking the CBD joint and from 42.0–77.5 (mean: 60.7) after consumption of the placebo joint. The consistency of the results was also apparent in the statistical analysis, revealing no significant diffe­rences in the comparison between CBD and placebo consumption, between male and female participants, or between the first and second trial. Thus, learning effects were excluded for the DT (Table 1).

#### Cognitrone test

The *t*-values for the average time of correct rejections ranged from 57.0–77.5 for both the CBD and placebo conditions. Although the mean values differed slightly between conditions (72.2 *versus* 71.7 after smoking the CBD and placebo joints, respectively), the analysis revealed no significant diffe­rences between smoking CBD-rich marijuana and placebo. Furthermore, no significant differences were detected when comparing between men and women, or between the first and second trials. Accordingly, learning effects were excluded for the Cognitrone test (Table 1).

### Tests for balance and coordination

#### Romberg test

The estimated time ranged from 25–68 s (mean: 34 s) after consumption of the CBD joint and from 26–55 s (mean: 37 s) after smoking the placebo joint. In both groups, the average value was slightly greater than the target value. Although this difference was statistically significant (*P *= 0.039), the results were well within the normal range of 20–45 s. Furthermore, no significant difference was detected between men and women (*P* = 0.913 for placebo and *P* = 0.648 for CBD) or between the first and second trials (*P* = 0.169). Thus, learning effects were excluded. In addition, a secure balance was observed in all participants, regardless of whether the CBD or placebo joint was smoked.

#### Finger-to-Nose test

Of the 33 participants, 32 touched the tip of the nose with each action. In addition, movements were undisturbed for 32 of 33 participants, regardless of whether the CBD or placebo joint was smoked. After consumption of the CBD joint, one participant missed the tip of his/her nose twice. Another participant’s movement paused when changing from right to right again, after smoking the placebo joint. The motion sequence (left-right-left-right-right-left) was performed incorrectly five times out of 66: three times after smoking the CBD joint and two times after smoking the placebo joint.

#### Walk-and-Turn test

None of the participants exhibited interrupted walking, and no participants missed the heel-to-toe or hop around, regardless of whether they smoked the CBD or placebo joint. Some discrepancies, however, were observed in the rotation and the number of steps taken. In total, the rotation was performed incorrectly eight times, three of which occurred after consuming the CBD joint. Furthermore, in 19 of 66 cases, an incorrect number of steps was taken. Again, these deviations were spread over both groups: the errors occurred 10 times after smoking the CBD joint and nine times after consumption of the placebo joint.

### Vital signs and observed changes in behaviour, orientation, mood, language and psychomotor skills

Systolic blood pressure ranged from 88–152 mmHg (mean: 128 mmHg) and from 106–169 mmHg (mean: 130 mmHg) after consumption of the CBD and the placebo joints, respectively. The diastolic blood pressure ranged from 57–106 mmHg (mean: 83 mmHg) and from 66–100 mmHg (mean: 82 mmHg) after smoking the CBD and the placebo joints, respectively. The pulse ranged from 58–115 /min (mean: 80 /min) after consumption of the CBD joint and from 61–117 /min (mean: 78 /min) after smoking the placebo joint.

No other changes in behaviour, orientation, mood, language and psychomotor skills were observed.

### Cannabinoid concentrations in capillary blood

Directly after smoking the CBD joint, free CBD and THC capillary blood concentrations were in the range of 2.6–440.0 ng/mL (mean: 126.0 ng/mL) and 6.7–102.0 ng/mL (mean: 28.7 ng/mL), respectively.

After completing all the tests, approximately 45 min later on average, free CBD and THC capillary blood concentrations were still in the range of 1.9–135.0 ng/mL (mean: 42.7 ng/mL) and 0.9–38.0 ng/mL (mean: 6.5 ng/mL), respectively.

11-Hydroxy-Δ^9^-THC (11-OH-THC), an active metabolite of THC, was only detected in six samples and in very low concentrations (<5 ng/mL). The inactive main metabolite, 11-nor-9-carboxy-Δ^9^-THC (THC-COOH) was not detected in any samples.

## Discussion

### Traffic psychological assessment

The traffic psychological assessment revealed similar distributions of results regardless of whether CBD-rich marijuana or placebo was smoked. All mean values were located above average zone, except for motor time, where the mean values were in the average zone. Of 198 tests, only three (1.5%) resulted in a *t*-value below the average zone and thus failed a test. During the reaction test, one participant achieved a *t*-value of 38, both after CBD and placebo consumption, but no irregularities were shown in other tests. Another participant failed the determination test after smoking CBD-rich marijuana. However, this occurred during the first trial, possibly because the participant was visibly nervous and insecure in the test environment. However, the parti­cipant passed the test on the second test day, although his/her test was still close to failing (*t*-value after CBD: 37.5 *vs.* 42.0 after placebo consumption).

The analysis only revealed significant differences in reaction time and motor time between men and women (Table 1). Because gender-related differences in reaction time were only observed after CBD consumption, it is possible that CBD had a stronger influence on women than men. In contrast, signifi­cant gender-related differences in motor time were found after both placebo and CBD consumption. These differences were more likely to be due to gender variance itself, and various studies have reported that men generally have faster reaction times and/or motor times than women [51].

Overall, no significant effects of CBD-rich mari­juana on driving ability were observed in the traffic psychological assessment of reaction time, motor time, stress resistance, or ability to concentrate.

### Balance and coordination tests

#### Romberg test

Of the 66 measurements, six (9.1%) were outside the normal range of 20–45 s. After smoking CBD-rich marijuana, in one case, the participant opened his/her eyes after 68 s, indicating a disturbed perception of time. However, this participant showed no irregu­larities in any other measurements, raising the possibility that the instructions were not clearly understood. According to Grubb’s outlier test, this result was an outlier (*G =* 4.32; critical *G*-value for sample size 35 is 2.82 with *α* = 0.05) and could therefore be rejected. Interestingly, the other five results outside the normal range occurred after smoking placebo. However, Grubb’s test indicated that these cases are not outliers and must therefore be retained (*G =* 1.27, 1.27, 1.83, 1.97 and 2.54). It should also be noted that all six cases outside the normal range occurred during the participants’ first attempt, and all cases were within the normal range after the second trial. Although this may have indicated a learning effect, statistical evaluation revealed no significant differences between the first and second trial results. Overall, smoking CBD-rich marijuana revealed no significant influence on performance in the Romberg test.

#### Finger-to-Nose test

The discrepancies observed during this test are likely to be due to divided attention, regardless of whether CBD-rich marijuana or placebo was smoked. It should be noted that the sequence itself did not appear to be the issue: in three cases, the participants simply started with the wrong hand (*right*-left-right-left-left-right) and in another case, an additional movement was made at the end with the right hand (left-right-left-right-right-left-*right*). In the fifth case, the arms were not returned to the starting position each time. However, the instructions regarding this point may have been insufficient. Overall, the results revealed no effect of CBD consumption on performance of the Finger-to-Nose test.

#### Walk-and-Turn test

The discrepancies observed in the current results are in accord with those reported in a previous study by Papafotiou et al. [52]. In that study, incorrect rotation and incorrect number of steps were reported to occur almost as frequently with the use of placebo as with the use of THC-rich marijuana, suggesting that performance was likely to be independent of drug use. Thus, the inclusion of these variables may lead to a high incidence of false-positive results. Further investigations are ne­cessary to determine whether these variables should be excluded from the assessment procedure. Considering these factors, it can be concluded that no influence on the performance of the Walk-and-Turn test was observed after smoking CBD-rich marijuana.

### Vital signs

No statistical evaluation of the vital signs was carried out because the measurement uncertainty of the device was not known, and only one measurement was taken per participant because of time constraints. Furthermore, the inclusion of gender variance itself was not feasible in the current study, and study participation itself can influence these parameters because it is not comparable to smoking marijuana in a recreational environment. Overall, the current results revealed no significant influence of smoking CBD-rich marijuana on vital signs. These observations are in accord with the results of a previous study by Bergamaschi et al. [29].

### Cannabinoid concentrations in capillary blood

During the current pilot study, 33 participants smoked a single joint of a 1:1 mixture of tobacco and legal CBD-rich marijuana *ad libitum* within 10 min, simu­lating acute use. Accurate estimation of the bioavai­lable amount of CBD and THC is difficult when administering cannabis by smoking. Various factors, such as varying inhalation efficiency and losses due to side-stream smoke and pyrolysis, must be taken into consideration. These factors are not controllable, making the estimation highly variable. Huestis [53] established that the bioavailability of CBD and THC ranges between 11%–45% and 10%–50%, respectively. Furthermore, losses due to pyrolytic destruction and side-stream smoke accounted for up to 30% and 50% of variation, respectively. The CBD-rich joints in our study contained 83 mg of CBD and 4.5 mg of THC. On the basis of these findings, approximately 58 mg of CBD and 3.2 mg of THC were likely to have been released by smoking, and an estimated dose of 29 mg of CBD and 1.6 mg of THC was likely to have been inhaled. With an average bioavailability of approximately 30%, the estimated systemically available amounts after smoking CBD-rich marijuana were 8.7 mg CBD and 0.5 mg THC.

The analyses of capillary blood samples directly after smoking resulted in detectable free CBD concentrations in all samples, ranging from 2.6–440.0 ng/mL (mean: 126.0 ng/mL). Free THC was detected in all but one sample and the THC positive cases ranged between 6.7–102.0 ng/mL (mean: 28.7 ng/mL), exceeding the cut-off value for driving under the influence of cannabis according to the Swiss Road Traffic regulations. The broad distribution between the lowest capillary blood concentrations (6.7 ng/mL free THC and 2.6 ng/mL free CBD; free CBD/THC ratio: 0.39) and the highest concentrations (102.0 ng/mL free THC and 440.0 ng/mL free CBD; free CBD/THC ratio: 4.31) could be explained by inter-individual differences in puff duration, inhaled smoke volume and breath-holding after inhalation [54]. Similar observations between occasional and heavy users have been conducted with participants smoking THC-rich marijuana, and Huestis et al. [55] reported peak levels ranging from 76.0 to 267.0 ng/mL. Even within the two groups of occasional and heavy consumers, inter-individual diffe­rences have been found, with Toennes et al. [56] reporting concentrations ranging from 11.9 to 86.0 ng/mL and 7.9 to 244.8 ng/mL for occasional and heavy users, respectively, after smoking a standardised THC joint.

In the capillary blood samples taken after the tests (approximately 50 min after smoking), free CBD was still detected in all samples, ranging between 1.9–135.0 ng/mL (mean: 42.7 ng/mL) and THC in 30 out of 33 samples (0.9–38.0 ng/mL; mean: 6.5 ng/mL). Compared with samples taken directly after smoking, the concentrations were three to four times lower on average. Of the 30 THC positive cases, only nine were below the cut-off value of 2.2 ng/mL. Accordingly, approximately 65% of all participants had blood concentrations of THC above the decision limit for driving under the influence of cannabis from a legal point of view even 45 min after the end of smoking CBD-rich marijuana. A comparison of the capillary blood concentrations with other studies is difficult because pharmacokinetic studies usually analysed venous whole blood or plasma. It is unclear at this stage whether there are differences in the concentrations of CBD and THC when comparing venous whole blood/plasma to capillary blood. This information is becoming more important as dried blood spot samples with capillary blood are increasingly widely used beyond forensic toxicology [57]. We are currently working on filling this knowledge gap. In another study in our laboratory, smoking experiments with CBD-rich marijuana were performed in which venous and capillary blood samples were taken simultaneously over a 60-min period (manuscript in preparation).

Considering that the tests performed in the current study revealed no signs of impairment, the high THC concentrations are surprising. Previous experi­mental studies of THC-rich marijuana have reported physiological impacts, including cardiovascular [58, 59] and neurological effects [60, 61]. Furthermore, it is known that THC influences certain psychologi­cal parameters, such as psychomotor performance (balance and body sway) [62–65], perception of passage of time [59] and more complex tasks requiring continuous monitoring and the ability to shift attention rapidly between various stimuli [66, 67]. Other studies [36–38, 52, 63] relating to driving ability show considerable reductions in certain parameters, with measured blood THC concentrations comparable to or even lower than those observed in our study. Similar observations have also been made in previous studies involving driving tests [40–42]. While the measured THC concentrations in these previous studies do not differ greatly from our study, they are associated with substantially different effects. Moreover, epidemiological studies by Drummer et al. [68] and Laumon et al. [69] reported that drivers with blood THC concentrations of 5 ng/mL or higher had a significantly higher likelihood of causing a crash than drug-free drivers, suggesting an impaired ability to drive. Similar results were also reported by Khiabani et al. [70], revealing that drivers with blood THC concentrations above 3 ng/mL had an increased risk of being judged as impaired.

The question therefore arises regarding why the current results revealed no signs of impairment despite elevated blood THC concentrations. Previous studies have suggested a possible protective effect of CBD, reporting that CBD can counteract the negative effects of THC [71]. However, the mechanisms by which CBD exerts these effects remain to be elucidated. Zuardi et al. [30] have shown that the administration interval and dose ratio between CBD and THC play an important role for the interaction of the two substances. An earlier study conducted by Zuardi et al. [72] and a more recent study by Bhattacharyya et al. [73] suggest that simultaneous administration of a high dose ratio of CBD/THC could favour a pharmacodynamic interaction over a pharmacokinetic interaction in humans, whereby an antagonistic effect occurs when the CBD/THC ratio is at least 8.1 (±11.1) [74]. This may also explain why no symptoms of impairment were observed during the tests in the current study, despite high blood THC concentrations. The total CBD concentration in the smoked marijuana was approximately 18 times higher than the total THC concentration. This could potentially explain why a recent study by Arkell et al. [39] reported that no preventative mechanisms of CBD were observed. The pharmacodynamic mechanisms responsible for the wide range of effects of CBD are currently unclear. It was traditionally presumed that CBD behaves like a first-generation CB_1_ and CB_2_ receptor inverse agonist [75, 76]. However, recent investigations suggest that CBD acts through negative allosteric mechanisms at these two receptors [77–79].

## Conclusion

In view of the legal availability of CBD-rich marijuana, we conducted a pilot study with 33 participants to investigate different psychomotor and neurocognitive skills with regard to driving ability. The traffic psychological assessment with three standardised and validated tests showed that there were no significant differences between smoking CBD-rich marijuana and placebo in reaction time, motor time, behaviour under stress, or concentration. Furthermore, balance and coordination tests indicated no significant differences.

These results are surprising because high THC concentrations were detected in capillary blood after smoking CBD-rich marijuana. Mean values ± SD were (28.7 ± 24.1) ng/mL directly after smoking and (6.5 ± 8.5) ng/mL after performing the tests. Although free THC concentrations reached levels that were considered to cause symptoms of impairment in other studies in which THC-rich marijuana was smoked, no signs of impairment were observed in the current study. These findings suggest that higher CBD concentrations caused a negative allosteric effect in the endocannabinoid system, preventing the formation of such symptoms.

To the best of our knowledge, this is one of the first studies investigating the potential impact of smoking CBD-rich marijuana on road safety. Although no symptoms of impairment were observed, it is recommended that consumers refrain from driving for several hours after smoking CBD-rich marijuana, as legal THC concentration limits may be exceeded.

## Limitations

In the current study, only six tests (four of which are validated and standardised) were performed, limi­ting the conclusions that can be drawn. Thus, it is possible that CBD has an impact on other characteristics of driving ability that were not tested in the current study. However, we believe that these six tests provide relatively comprehensive results.

Furthermore, the absence of a positive control (e.g. with THC-rich marijuana) should be considered when interpreting the current results. This lack of a control condition is based on the decisions of the responsible ethics committee. This was also the case regarding the inclusion of other examinations, such as on-the-road driving performance tests.

## Supplementary Material

Supplemental Material

## References

[1] UNODC. World Drug Report 2019. Vienna (Austria): UNODC. 2019. [cited 2020 March 10th]. Available from: https://wdr.unodc.org/wdr2019/

[2] Gmel G, Kuendig H, Notari L, et al. Suchtmonitoring Schweiz – Konsum von Alkohol, Tabak und illegalen Drogen in der Schweiz im Jahr 2016. Lausanne (Switzerland): Sucht Schweiz; 2017. German.

[3] Castle D, Murray R, editors. Marijuana and madness: psychiatry and neurobiology. Cambridge (UK): Cambridge University Press; 2004.

[4] Chandra S, Lata H, ElSohly MA, editors. *Cannabis sativa* L. – Botany and Biotechnology. Cham (Switzerland): Springer International Publishing; 2017.

[5] Cheng D, Spiro AS, Jenner AM, et al. Long-term cannabidiol treatment prevents the development of social recognition memory deficits in Alzheimer’s disease transgenic mice. J Alzheimers Dis. 2014;42:1383–1396. 25024347 10.3233/JAD-140921

[6] Ben-Menachem EA-O, Gunning B, Arenas Cabrera CM, et al. A phase II randomized trial to explore the potential for pharmacokinetic drug-drug interactions with stiripentol or valproate when combined with cannabidiol in patients with epilepsy. CNS Drugs. 2020;34:661–672. 32350749 10.1007/s40263-020-00726-4 PMC7275018

[7] Bialer M, Perucca E. Does cannabidiol have antiseizure activity independent of its interactions with clobazam? An appraisal of the evidence from randomized controlled trials. Epilepsia. 2020;61:1082–1089. 32452568 10.1111/epi.16542

[8] Cross JH, Cock H. A perspective on cannabinoids for treating epilepsy: do they really change the landscape? Neuropharmacology. 2020;170:107861. 31770546 10.1016/j.neuropharm.2019.107861

[9] Devinsky O, Marsh E, Friedman D, et al. Cannabidiol in patients with treatment-resistant epilepsy: an open-label interventional trial. Lancet Neurol. 2016;15:270–278. 26724101 10.1016/S1474-4422(15)00379-8

[10] Galan FN, Miller I. Cannabinoids for the treatment of epilepsy: a review. Curr Treat Options Neurol. 2020;22:14.

[11] Herlopian A, Hess EJ, Barnett J, et al. Cannabidiol in treatment of refractory epileptic spasms: an open-label study. Epilepsy Behav. 2020;106:106988. 32169600 10.1016/j.yebeh.2020.106988

[12] Miller I, Scheffer IE, Gunning B, et al. Dose-ranging effect of adjunctive oral cannabidiol *vs* placebo on convulsive seizure frequency in dravet syndrome: a randomized clinical trial. JAMA Neurol. 2020;77:613–621. 32119035 10.1001/jamaneurol.2020.0073 PMC7052786

[13] Puteikis K, Mameniškienė R. Use of cannabis and its products among patients in a tertiary epilepsy center: a cross-sectional survey. Epilepsy Behav. 2020;111:107214. 32580133 10.1016/j.yebeh.2020.107214

[14] von Wrede R, Moskau-Hartmann S, Amarell N, et al. Plant derived versus synthetic cannabidiol: wishes and commitment of epilepsy patients. Seizure. 2020;80:92–95. 32554292 10.1016/j.seizure.2020.06.012

[15] Alessandria G, Meli R, Infante MT, et al. Long-term assessment of the cognitive effects of nabiximols in patients with multiple sclerosis: a pilot study. Clin Neurol Neurosurg. 2020;196:105990. 32526487 10.1016/j.clineuro.2020.105990

[16] Calabrò RS, Russo M, Naro A, et al. Nabiximols plus robotic assisted gait training in improving motor performances in people with Multiple Sclerosis. Mult Scler Relat Disord. 2020;43:102177. 32447249 10.1016/j.msard.2020.102177

[17] Giacoppo S, Rajan TS, Galuppo M, et al. Purified Cannabidiol, the main non-psychotropic component of *Cannabis sativa*, alone, counteracts neuronal apoptosis in experimental multiple sclerosis. Eur Rev Med Pharmacol Sci. 2015;19:4906–4919. 26744883

[18] Maayah ZH, Takahara S, Ferdaoussi M, et al. The anti-inflammatory and analgesic effects of formulated full-spectrum cannabis extract in the treatment of neuropathic pain associated with multiple sclerosis. Inflamm Res. 2020;69:549–558. 32239248 10.1007/s00011-020-01341-1

[19] Ghabrash MF, Coronado-Montoya S, Aoun J, et al. Cannabidiol for the treatment of psychosis among patients with schizophrenia and other primary psychotic disorders: a systematic review with a risk of bias assessment. Psychiatry Res. 2020;286:112890. 32126328 10.1016/j.psychres.2020.112890

[20] Gomes FV, Llorente R, Del Bel EA, et al. Decreased glial reactivity could be involved in the antipsychotic-like effect of cannabidiol. Schizophr Res. 2015;164:155–163. 25680767 10.1016/j.schres.2015.01.015

[21] Rodrigues da Silva N, Gomes FV, Sonego AB, et al. Cannabidiol attenuates behavioral changes in a rodent model of schizophrenia through 5-HT1A, but not CB1 and CB2 receptors. Pharmacol Res. 2020;156:104749. 32151683 10.1016/j.phrs.2020.104749

[22] Schoevers J, Leweke JE, Leweke FM. Cannabidiol as a treatment option for schizophrenia: recent evidence and current studies. Curr Opin Psychiatry. 2020;33:185–191. 32073423 10.1097/YCO.0000000000000596

[23] Kovalchuk O, Kovalchuk I. Cannabinoids as anticancer therapeutic agents. Cell Cycle. 2020;19:961–989. 32249682 10.1080/15384101.2020.1742952 PMC7217364

[24] Rocha FCM, Dos Santos Júnior JG, Stefano SC, et al. Systematic review of the literature on clinical and experimental trials on the antitumor effects of cannabinoids in gliomas. J Neurooncol. 2014;116:11–24. 24142199 10.1007/s11060-013-1277-1

[25] Iffland K, Grotenhermen F. An update on safety and side effects of cannabidiol: a review of clinical data and relevant animal studies. Cannabis Cannabinoid Res. 2017;2:139–154. 28861514 10.1089/can.2016.0034 PMC5569602

[26] Gertsch J. The intricate influence of the placebo effect on medical cannabis and cannabinoids. Med Cannabis Cannabinoids. 2018;1:60–64. 34676323 10.1159/000489291 PMC8489322

[27] Mechoulam R, Parker LA, Gallily R. Cannabidiol: an overview of some pharmacological aspects. J Clin Pharmacol. 2002;42:11S–19S. 12412831 10.1002/j.1552-4604.2002.tb05998.x

[28] Pisanti S, Malfitano AM, Ciaglia E, et al. Cannabidiol: state of the art and new challenges for therapeutic applications. Pharmacol Ther. 2017;175:133–150. 28232276 10.1016/j.pharmthera.2017.02.041

[29] Bergamaschi MM, Queiroz RHC, Zuardi AW, et al. Safety and side effects of cannabidiol, a *Cannabis sativa* constituent. Curr Drug Saf. 2011;6:237–249. 22129319 10.2174/157488611798280924

[30] Zuardi AW, Hallak JEC, Crippa JAS. Interaction between cannabidiol (CBD) and Δ9-tetrahydrocannabinol (THC): influence of administration interval and dose ratio between the cannabinoids. Psychopharmacology (Berl). 2012;219:247–249. 21947314 10.1007/s00213-011-2495-x

[31] Jorio L. Überproduktion von legalem Cannabis lässt Hanf-Exporte anschwellen. 2018. [cited 2021 June 15]. Available from: https://www.swissinfo.ch/ger/cbd-hanf_ueberproduktion-von-legalem-cannabis-laesst-hanf-exporte-anschwellen/44269370. German.

[32] Delgrande Jordan M, Schneider E, Eichenberger Y, et al. La consommation de substances psychoactives des 11 à 15 ans en Suisse – Situation en 2018 et évolutions depuis 1986 – Résultats de l’étude Health Behaviour in Shool-aged Children (HSBC). Lausanne (Switzerland): Addiction Suisse; 2019. French.

[33] Hädener M, Gelmi TJ, Martin-Fabritius M, et al. Cannabinoid concentrations in confiscated cannabis samples and in whole blood and urine after smoking CBD-rich cannabis as a “tobacco substitute”. Int J Legal Med. 2019;133:821–832. 30612324 10.1007/s00414-018-01994-y

[34] Meier U, Dussy F, Scheurer E, et al. Cannabinoid concentrations in blood and urine after smoking cannabidiol joints. Forensic Sci Int. 2018;291:62–67. 30149280 10.1016/j.forsciint.2018.08.009

[35] Spindle TR, Cone EJ, Goffi E, et al. Pharmacodynamic effects of vaporized and oral cannabidiol (CBD) and vaporized CBD-dominant cannabis in infrequent cannabis users. Drug Alcohol Depend. 2020;211:107937. 32247649 10.1016/j.drugalcdep.2020.107937 PMC7414803

[36] Böcker KBE, Gerritsen J, Hunault CC, et al. Cannabis with high Δ9-THC contents affects perception and visual selective attention acutely: an event-related potential study. Pharmacol Biochem Behav. 2010;96:67–74. 20417659 10.1016/j.pbb.2010.04.008

[37] Ménétrey A, Augsburger M, Favrat B, et al. Assessment of driving capability through the use of clinical and psychomotor tests in relation to blood cannabinoids levels following oral administration of 20 mg dronabinol or of a cannabis decoction made with 20 or 60 mg Δ9-THC. J Anal Toxicol. 2005;29:327–338. 16105257 10.1093/jat/29.5.327

[38] Ramaekers JG, Moeller MR, van Ruitenbeek P, et al. Cognition and motor control as a function of Δ9-THC concentration in serum and oral fluid: li­mits of impairment. Drug Alcohol Depend. 2006;85:114–122. 16723194 10.1016/j.drugalcdep.2006.03.015

[39] Arkell TR, Lintzeris N, Kevin RC, et al. Cannabidiol (CBD) content in vaporized cannabis does not prevent tetrahydrocannabinol (THC)-induced impairment of driving and cognition. Psychopharmacology. 2019;236:2713–2724. 31044290 10.1007/s00213-019-05246-8 PMC6695367

[40] Bosker WM, Kuypers KPC, Theunissen EL, et al. Medicinal Δ9 -tetrahydrocannabinol (dronabinol) impairs on-the-road driving performance of occasional and heavy cannabis users but is not detected in Standard Field Sobriety Tests. Addiction. 2012;107:1837–1844. 22553980 10.1111/j.1360-0443.2012.03928.x

[41] Ramaekers JG, Robbe HWJ, O’Hanlon JF. Marijuana, alcohol and actual driving performance. Hum Psychopharmacol Clin Exp. 2000;15:551–558. 10.1002/1099-1077(200010)15:7<551::AID-HUP236>3.0.CO;2-P 12404625

[42] Robbe HW, O’Hanlon JF. Marijuana and actual driving performance. Springfield (VA): US Department of Transportation, National Highway Traffic Safety Administration; 1993. DOT HS 808 078.

[43] Ambach L, Penitschka F, Broillet A, et al. Simultaneous quantification of Δ9-THC, THC-acid A, CBN and CBD in seized drugs using HPLC-DAD. Forensic Sci Int. 2014;243:107–111. 25005819 10.1016/j.forsciint.2014.06.008

[44] Talpins SK, Hayes C. The Drug Evaluation and Classification (DEC) program – targetting hardcore impaired drivers. Alexandria (VA): American Prosecutors Research Institute; 2004.

[45] Hauri-Bionda R, Friedrich-Koch A, Herrmann PW. MEDRALEX für Ärzte – Eine moderne Strategie zum medizinischen Nachweis von Fahrunfähigkeit. Zurich (Switzerland): Institute of Forensic Medicine, University of Zürich; 1998. German.

[46] Sponsel R. Prozent *RANG*— Bedeutung, Berechnung, problemlösung, Literatur, Links. German.

[47] Schubert W, Dittmann V, Brenner-Hartmann J. Urteilsbildung in der Fahreignungsbegutachtung –Beurteilungskriterien. 3rd ed. Bonn (Germany): Kirschbaum Verlag; 2013.

[48] Schuhfried-GmbH. Wiener German. test system manual: fitness to drive plus [DRIVEPLS]. Version 04, Revision 6. 2018.

[49] Lauth GW, Grünke M, Brunstein JC, editors. Interventionen bei Lernstörungen. 2nd, revised and extended. ed. Göttingen (Germany): Hogrefe; 2014. German.

[50] König S, Aebi B, Lanz S, et al. On-line SPE LC-MS/MS for the quantification of Δ9-tetrahydrocannabinol (THC) and its two major metabolites in human peripheral blood by liquid chromatography tandem mass spectrometry. Anal Bioanal Chem. 2011;400:9–16. 21311875 10.1007/s00216-011-4708-x

[51] Der G, Deary I. Age and sex differences in reaction time in adulthood: results from the United Kingdom Health and Lifestyle Survey. Psychol Aging. 2006;21:62–73. 16594792 10.1037/0882-7974.21.1.62

[52] Papafotiou K, Carter JD, Stough C. An evaluation of the sensitivity of the Standardised Field Sobriety Tests (SFSTs) to detect impairment due to marijuana intoxication. Psychopharmacology (Berl). 2005;180:107–114. 15619106 10.1007/s00213-004-2119-9

[53] Huestis MA. Pharmacokinetics and metabolism of the plant cannabinoids, Δ9-tetrahydrocannibinol, cannabidiol and cannabinol. In: Pertwee RG, editor. Cannabinoids. Handbook of experimental pharmacology. Vol. 168. Heidelberg (Germany): Springer Berlin, Heidelberg; 2005. 10.1007/3-540-26573-2_23 16596792

[54] Agurell S, Halldin M, Lindgren JE, et al. Pharmacokinetics and metabolism of Δ1-tetrahydrocannabinol and other cannabinoids with emphasis on man. Pharmacol Rev. 1986;38:21. 3012605

[55] Huestis MA, Henningfield JE, Cone EJ. Blood cannabinoids. I. Absorption of THC and formation of 11-OH-THC and THCCOOH during and after smoking marijuana. J Anal Toxicol. 1992;16:276–282. 1338215 10.1093/jat/16.5.276

[56] Toennes SW, Ramaekers JG, Theunissen EL, et al. Comparison of cannabinoid pharmacokinetic pro­perties in occasional and heavy users smoking a mari­juana or placebo joint. J Anal Toxicol. 2008;32:470–477. 18713514 10.1093/jat/32.7.470

[57] Demirev PA. Dried blood spots: analysis and applications. Anal Chem. 2013;85:779–789. 23171435 10.1021/ac303205m

[58] Jones RT. Cardiovascular system effects of marijuana. J Clin Pharmacol. 2002;42:58S–63S. 12412837 10.1002/j.1552-4604.2002.tb06004.x

[59] Logan BK. Marijuana and driving impairment. In: ElSohly MA, editor. Marijuana and the cannabinoids. Forensic Science and Medicine. Totowa (NJ): Humana Press; 2007.

[60] Macdonald S, Anglin-Bodrug K, Mann RE, et al. Injury risk associated with cannabis and cocaine use. Drug Alcohol Depend. 2003;72:99–115. 14636965 10.1016/s0376-8716(03)00202-3

[61] Wilkins JN, Mellot KG, Markvitsa R, et al. Management of stimulant, hallucinogen, marijuana, phencyclidine, and club drug intoxication and withdrawl. In: Graham AW, Schultz TK, Mayo-Smith MF, et al., editors. Principles of addiction medicine. Chevy Chase (MD): American Society of Addiction Medicine; 2003. p. 671–695.

[62] Ford TC, Hayley AC, Downey LA, et al. Cannabis: an overview of its adverse acute and chronic effects and its implications. Curr Drug Abuse Rev. 2017;10:6–18. 28707583 10.2174/1874473710666170712113042

[63] Greenberg HS, Werness SAS, Pugh JE, et al. Short-term effects of smoking marijuana on balance in patients with multiple sclerosis and normal volu­nteers. Clin Pharmacol Ther. 1994;55:324–328. 8143398 10.1038/clpt.1994.33

[64] Heishman SJ. Effects of marijuana on human performance and assessment of driving impairment. In: Onaivi ES, editor. The biology of marijuana — from gene to behavior. London (UK): CRC Press; 2002.

[65] Liguori A, Gatto CP, Robinson JH. Effects of mari­juana on equilibrium, psychomotor performance, and simulated driving. Behav Pharmacol. 1998;9:599–609. 9862085 10.1097/00008877-199811000-00015

[66] Hart CL, Ward AS, Haney M, et al. Comparison of smoked marijuana and oral Δ9-tetrahydrocannabinol in humans. Psychopharmacology (Berl). 2002;164:407–415. 12457271 10.1007/s00213-002-1231-y

[67] Kelly TH, Foltin RW, Emurian CS, et al. Performance-based testing for drugs of abuse: dose and time profiles of marijuana, amphetamine, alcohol, and diazepam. J Anal Toxicol. 1993;17:264–272. 8107459 10.1093/jat/17.5.264

[68] Drummer OH, Gerostamoulos J, Batziris H, et al. The involvement of drugs in drivers of motor vehicles killed in Australian road traffic crashes. Accid Anal Prev. 2004;36:239–248. 14642878 10.1016/s0001-4575(02)00153-7

[69] Laumon B, Gadegbeku B, Martin J-L, et al. Cannabis intoxication and fatal road crashes in France: popu­lation based case-control study. BMJ. 2005;331:1371. 16321993 10.1136/bmj.38648.617986.1F PMC1309644

[70] Khiabani HZ, Bramness JRG, Bjørneboe A, et al. Relationship between THC concentration in blood and impairment in apprehended drivers. Traffic Inj Prev. 2006;7:111–116. 16854704 10.1080/15389580600550172

[71] Niesink RJM, van Laar M. Does cannabidiol protect against adverse psychological effects of THC? Front Psychiatry. 2013;4:130. 24137134 10.3389/fpsyt.2013.00130 PMC3797438

[72] Zuardi AW, Shirakawa I, Finkelfarb E, et al. Action of cannabidiol on the anxiety and other effects produced by Δ9-THC in normal subjects. Psychopharmacology (Berl). 1982;76:245–250. 6285406 10.1007/BF00432554

[73] Bhattacharyya S, Morrison PD, Fusar-Poli P, et al. Opposite effects of Δ9-tetrahydrocannabinol and cannabidiol on human brain function and psychopathology. Neuropsychopharmacology. 2010;35:764–774. 19924114 10.1038/npp.2009.184 PMC3055598

[74] Zuardi AW, Teixeira NA, Karniol IC. Pharmacological interaction of the effects of Δ9-trans-tetrahydrocannabinol and cannabidiol on serum corticosterone levels in rats. Arch Int Pharmacodyn Ther. 1984;269:12–19. 6087750

[75] Pertwee RG. The diverse CB1 and CB2 receptor pharmacology of three plant cannabinoids: Δ9-tetrahydrocannabinol, cannabidiol and Δ9-tetrahydrocannabivarin. Br J Pharmacol. 2008;153:199–215. 17828291 10.1038/sj.bjp.0707442 PMC2219532

[76] Thomas A, Baillie GL, Phillips AM, et al. Cannabidiol displays unexpectedly high potency as an antagonist of CB1 and CB2 receptor agonists *in vitro*. Br J Pharmacol. 2007;150:613–623. 17245363 10.1038/sj.bjp.0707133 PMC2189767

[77] McPartland JM, Duncan M, Di Marzo V, et al. Are cannabidiol and Δ9 -tetrahydrocannabivarin ­negative modulators of the endocannabinoid system? A systematic review. Br J Pharmacol. 2015;172:737–753. 25257544 10.1111/bph.12944 PMC4301686

[78] Martínez-Pinilla E, Varani K, Reyes-Resina I, et al. Binding and signaling studies disclose a potential allosteric site for cannabidiol in cannabinoid CB2 receptors. Front Pharmacol. 2017;8:744. 29109685 10.3389/fphar.2017.00744 PMC5660261

[79] Tham M, Yilmaz O, Alaverdashvili M, et al. Allosteric and orthosteric pharmacology of cannabidiol and cannabidiol-dimethylheptyl at the type 1 and type 2 cannabinoid receptors. Br J Pharmacol. 2019;176:1455–1469. 29981240 10.1111/bph.14440 PMC6487556

