# Supplementary material for: Impact of smoking cannabidiol (CBD)-rich marijuana on driving ability
Source: Forensic Sci Res. 2021 Sep 28;6(3):195–207. doi: 10.1080/20961790.2021.1946924 (PMC8635612; doi:10.1080/20961790.2021.1946924)
Supplement: Supplemental Material [file TFSR_A_1946924_SM5918.zip › TFSR_A_1946924_supplementary_material/TFSR_A_1946924_Supplementary_material.docx]

**Impact of smoking CBD-rich marijuana on driving ability**

Authors:

Tim J. Gelmi^1,2^, Wolfgang Weinmann^1*^, Matthias Pfäffli^3^

Affiliations and Addresses:

^1^Institute of Forensic Medicine

Department of Forensic Toxicology and Chemistry

University of Bern

Bühlstrasse 20

3012 Bern

Switzerland

^2^Graduate School for Cellular and Biomedical Sciences (GCB)

University of Bern

Mittelstrasse 43

3012 Bern

Switzerland

^3^Institute of Forensic Medicine

Department of Traffic Sciences

University of Bern

Sulgenauweg 40

3007 Bern

Switzerland

*Corresponding author:

Prof. Dr. Wolfgang Weinmann

E-Mail address: [wolfgang.weinmann@irm.unibe.ch](mailto:wolfgang.weinmann@irm.unibe.ch)

Telephone number with country code: +41 (0)31 684 5668

ORCID:

Tim J. Gelmi 0000-0003-1684-4649

Wolfgang Weinmann 0000-0001-8659-1304

Matthias Pfäffli 0000-0003-2712-8672

**Supplementary Tables**

| **Table S1**. Participant's demographics | | | |
| --- | --- | --- | --- |
|  | Mean (SD) | | |
|  | Total | Male | Female |
| Sex (n°) | 33 | 19 | 14 |
| Age (years) | 24 (3) | 24 (3) | 24 (3) |
| Height (m) | 1.77 (0.09) | 1.82 (0.06) | 1.69 (0.06) |
| Weight (kg) | 73 (11) | 80 (7) | 64 (8) |
| BMI (kg/m^2^) | 23.4 (2.6) | 24.2 (2.2) | 22.3 (2.6) |
