# Supplementary figures and images for: Impact of smoking cannabidiol (CBD)-rich marijuana on driving ability
Source: Forensic Sci Res. 2021 Sep 28;6(3):195–207. doi: 10.1080/20961790.2021.1946924 (PMC8635612; doi:10.1080/20961790.2021.1946924)

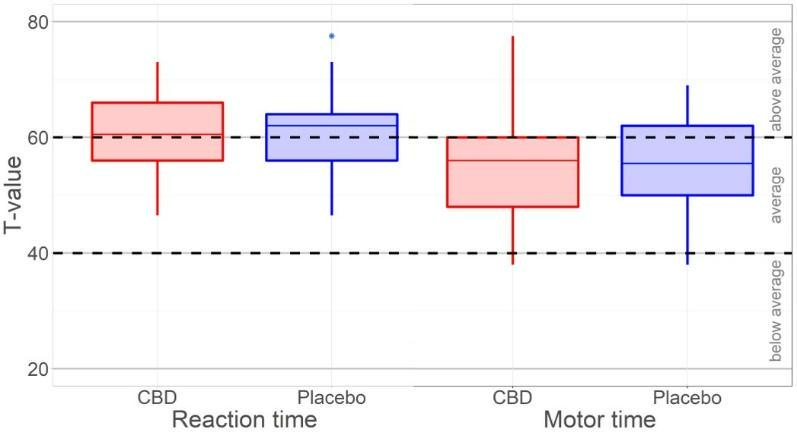

Supplement: Supplemental Material [file TFSR_A_1946924_SM5918.zip › TFSR_A_1946924_supplementary_material/Figure S1 of TFSR-2021-0045.png]

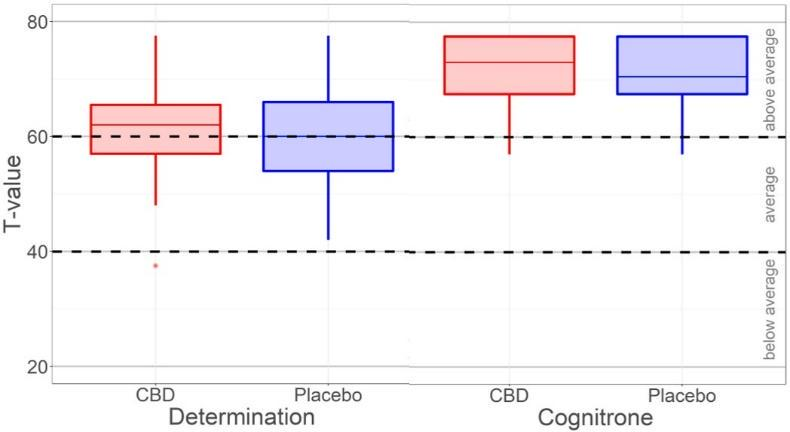

Supplement: Supplemental Material [file TFSR_A_1946924_SM5918.zip › TFSR_A_1946924_supplementary_material/Figure S2 of TFSR-2021-0045.png]

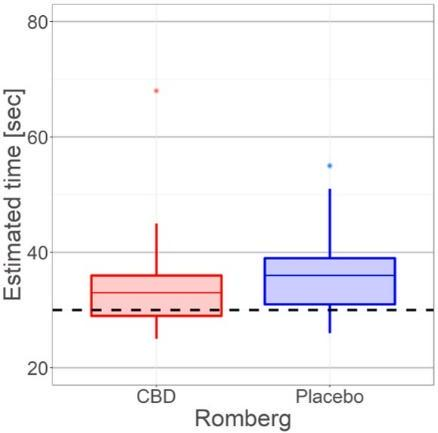

Supplement: Supplemental Material [file TFSR_A_1946924_SM5918.zip › TFSR_A_1946924_supplementary_material/Figure S3 of TFSR-2021-0045.png]

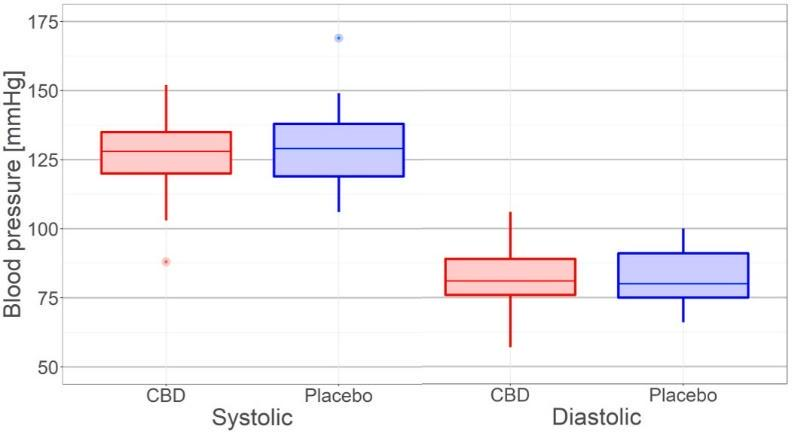

Supplement: Supplemental Material [file TFSR_A_1946924_SM5918.zip › TFSR_A_1946924_supplementary_material/Figure S4 of TFSR-2021-0045.png]

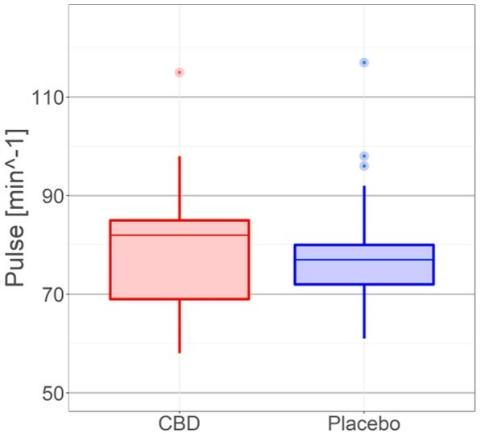

Supplement: Supplemental Material [file TFSR_A_1946924_SM5918.zip › TFSR_A_1946924_supplementary_material/Figure S5 of TFSR-2021-0045.png]

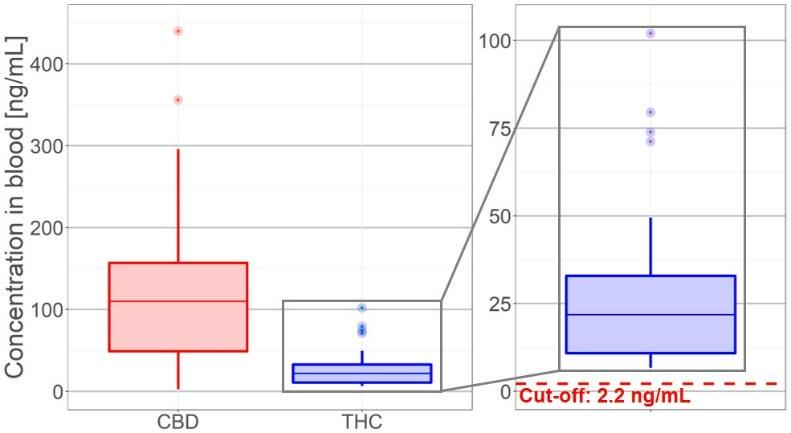

Supplement: Supplemental Material [file TFSR_A_1946924_SM5918.zip › TFSR_A_1946924_supplementary_material/Figure S6 of TFSR-2021-0045.png]

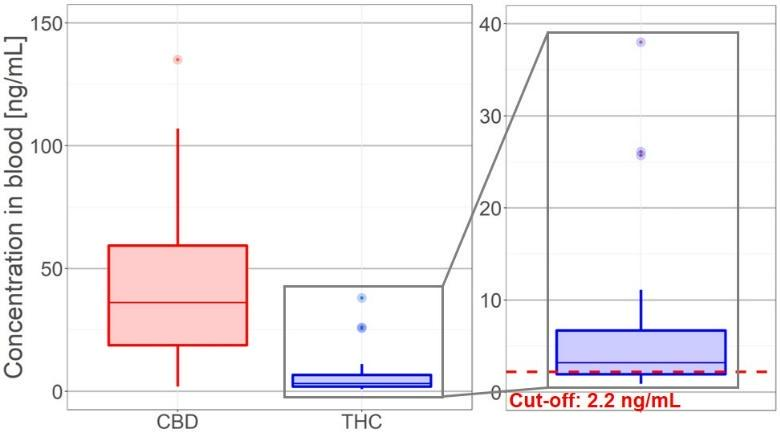

Supplement: Supplemental Material [file TFSR_A_1946924_SM5918.zip › TFSR_A_1946924_supplementary_material/Figure S7 of TFSR-2021-0045.png]
